# A systematic review study on the factors affecting shortage of nursing workforce in the hospitals

**DOI:** 10.1002/nop2.1434

**Published:** 2022-10-27

**Authors:** Adel Tutuo Tamata, Masoud Mohammadnezhad

**Affiliations:** ^1^ Vanuatu College of Nursing Education Ministry of Health Port Vila Vanuatu; ^2^ School of Nursing and Healthcare Leadership University of Bradford Bradford UK

**Keywords:** nurse burnout, nurse retention, nursing shortage, nursing workforce, systematic review

## Abstract

**Aim:**

This study aimed to determine factors that influence the nursing workforce shortage and their impact on nurses.

**Design:**

This study applied a systematic review design.

**Methods:**

Using Cochrane library guidelines, five electronic databases were systematically searched (Research *4*life—PubMed/Medline, Scopus, Embase, CINAHL) from 2010–2021. The remaining articles with pertinent information were presented in a data extraction sheet for further thematic analysis. A Reporting Items for Systematic Reviews and Meta‐Analysis Flow Diagram was adopted and used. The studies published from 2010–2021 and in English language were examined and included in the systematic review.

**Results:**

Four themes were identified as factors influencing the nursing workforce shortage, including Policy and planning barriers, Barriers to training and enrolment, Factors causing nursing staff turnover and Nurses' stress and burnout. Nursing workforce shortage is a global challenge that roots in multiple causes such as individual, educational, organizational and managerial and policy‐making factors.

## 1 INTRODUCTION

Nurses are essential workforce and form the largest professional group, which comprises healthcare workforce within the healthcare system in contributing significantly in delivering quality healthcare services, and assisting in the improvement of health outcomes for individuals, families and communities either through preventative and curative measures (Alameddine et al., 2017; Drennan & Ross, 2019). Furthermore, they are profoundly valued professionals and frontline healthcare workers in the world's healthcare system not only in delivering effective quality care but also in improving the efficiency of the health system; therefore, the adequate number of nurses is crucial to strengthen the health system to improve health coverage and achievements of all health targets (Aboshaiqah, 2016; Alameddine et al., 2017).

While the world has credibly acknowledged nursing profession as vital in delivering healthcare services, one of the main challenges faced today is the shortage of nursing workforce, which causes severe compromise on the quality of healthcare services, and in improving the well‐being of the global population and in achieving universal health coverage (Adams et al., 2021; Alameddine et al., 2017; Kurjenluoma et al., 2017; Park & Yu, 2019; Yahyaei et al., 2022). This is due to disproportions between the number of existing nurses and those that are entering the nursing profession compared with the required number of nurses to meet the patient's needs (Hudgins, 2016). Many studies discovered that there are discrepancies in the supply of nurses to maintain the adequate number of nursing workforce in response to ageing population, retirement along with the new recruitment of nurses as well as in retaining the existing nurses, which made it difficult to respond to the growing demand (Adams et al., 2021; Alameddine et al., 2017; Heijden et al., 2019; Matsuo et al., 2021; Otto et al., 2019; Park & Yu, 2019; Yahyaei et al., 2022). For instance, in the United States of America (USA), there is an extreme shortage of nurses as it will need approximately 3 million nurses to fulfil its nursing gap, a demand that cannot be easily achieved (Yun et al., 2010) and an estimate of 12.9 million deficit of skilled nurses and midwives by 2035 (Adams et al., 2021; Yahyaei et al., 2022). Furthermore, it is estimated that the shortage of nurses may exceed 500,000 by year 2025, while in Europe, 590,000 nursing vacancies are estimated by year 2020 (Valizadeh et al., 2016). Moreover, in South Korea, the shortage of nursing is very serious that the average number of nurses per 1,000 people is only 6.9 compared with the other organization for Economic Co‐operation and Development (OECD) member countries with 9.2 and above. Heijden et al. (2019) added that in most developed countries in European Union and others, the shortage of nurses in the next two decades is likely to worsen in response to the supply of nurses and the demand.

## 2 BACKGROUND

According to the World Health Organization (WHO) report, it was estimated that there will be a shortage of 7.2 million health workers to deliver healthcare services worldwide, and by 2035, the demand of nursing will reach 12.9 million (Adams et al., 2021). The impact of nursing workforce shortage is a huge challenge globally and is affecting more than one billion people, especially vulnerable populations such as women and children who badly needed the quality healthcare services (Aluko et al., 2019; Marć et al., 2019). The inadequate supply of nurses has notably created many negative impacts on the patient's health‐related outcome as well as challenges to fight diseases and improving health, which causes increased workload and stress levels on nurses and later results in decreasing the quality of nursing care, threatening the safety of patient and increasing the patient's mortality rate (Heijden et al., 2019; Leineweber et al., 2016; Matsuo et al., 2021; Valizadeh et al., 2016; Varasteh et al., 2021; Yun et al., 2010).

There are many factors affecting the healthcare system as a result of the shortage of nursing workforce. If these factors are not addressed promptly and appropriately, the number of people requiring quality nursing care will continue to be affected and the primary goal to improve and protect the health of the individual will continue to be a challenge (Matsuo et al., 2021; Valizadeh et al., 2016). These include the decreased number of student nurse's enrolment in nursing programme due to a lack of proper planning and funding availability and the increased number of early retirement due to health problem (Alameddine et al., 2017; Alshmemri et al., 2013; Barnett et al., 2010; Valizadeh et al., 2016). In Japan, the declining birth rate and increased population causes the inadequate number of workforce (Matsuo et al., 2021). Furthermore, in Thailand, the rapid migration of nurses due to poor working environment and conditions is the main cause of nursing shortage in the country (Nantsupawat et al., 2017). While in Singapore and other developed countries, job dissatisfaction remains the main reason for nurses' migration (Aeschbacher & Addor, 2018; Alshmemri et al., 2013; Hung & Lam, 2020; Leineweber et al., 2016). Furthermore, in Lebanon, the nursing workforce suffers due to the high migration rate of new graduating nurses with Bachelor's degree to other countries just few years after their graduation due to brain drain (Alameddine et al., 2017). However, one of the main factors reported in many countries is inadequate policies and workforce planning (Abhicharttibutra et al., 2017; Amadi, 2015; Marć et al., 2019; Mehdaova, 2017). Frequent shortage of nursing in a healthcare system to provide services may lead to stress and burnout, which will affect nurse's performance and increase the chances of medical errors, especially in patient's treatment, clinical care and laboratory tests (Aboshaiqah, 2016; Hung & Lam, 2020; Matsuo et al., 2021; Otto et al., 2019; Park & Yu, 2019; Varasteh et al., 2021).

More literatures on the shortage of nursing are from the western studies with only few from the Pacific island countries. It is important to get a better understanding on the specific factors that affect shortage in the region to have a meaningful strategy to resolve the current shortage of nursing workforce. The aim of this review is to explore the key factors that are most affecting the shortage of nursing workforce and to identify areas for future research.

## 3 METHODS

### 3.1 Search strategy

This systematic review study was conducted using Cochrane library guidelines. Five relevant databases were used to reach studies including Research *4*life—PubMed/Medline, Scopus, Embase, CINAHL using search terms applicable to specific databases. “Booleans” operator was also used in the search strategy such as (AND and OR) to acquire best information between the following keywords: “Nursing workforce,” “Nursing staff,” factors, determinants and shortage. These were used to locate relevant studies that explore the factors affecting the shortage of nursing workforce and the issues affecting nurses and patient health outcomes.

### 3.2 Selection criteria

All types of studies (qualitative, quantitative and mixed‐method) globally were considered in this review to extract the relevant articles among nurses in hospital settings and other health facilities. The studies published from 1 January 2010 to 31 August 2021 and in English language were examined and included from peer‐reviewed journals, published books and WHO reports with full text available that were related to the nursing workforce shortage and were suitable to support the current research study.

Systematic review studies and studies that their full text was not available were excluded. The information on shortage in other disciplines such as nurse aids, physicians and allied professionals are also excluded from this study.

### 3.3 Selection process

**FIGURE 1:**
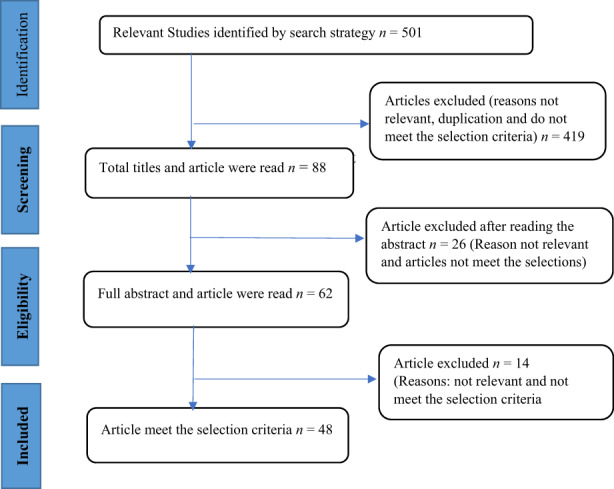
Article search and selection process

The selection process begins by checking all the titles and abstracts of the articles to identify pertinent articles. After the abstracts, the full texts on the remaining articles were checked to see whether they are applicable to the current research study. The articles were also checked for duplication articles or studies and only articles that suited the inclusion and exclusion criteria were downloaded and saved for use. The bibliography of all the remained studies was also checked to find some other articles that are published but were not in the selected databases. Moreover, 42 articles altogether were retrieved, which addressed the factors that influence the shortage in nursing workforce, its impact on registered nurses and the recommended interventions and measures to resolve the nursing workforce shortage. All the selected articles were then grouped under the major themes. A Preferred Reporting Items for Systematic Reviews and Meta‐Analysis (PRISMA) Flow Diagram in Figure 1 was adopted and used as a preferred reporting item for systematic reviews (Moher et al., 2009).

A data extraction sheet was developed to extract relevant information needed for further analysis and create themes for this study (Table 1).

**TABLE 1:** Data extraction sheet

N	Study information	Objective	Participants	Study design	Results
1	Aboshaiqah, 2016 Saudi Arabia	To investigate the nursing shortage in Saudi Arabia healthcare workforce.	Nurses in Saudi Arabia from 1993 to 2013	reviewing literature on the nursing shortage	‐ Nursing supply depends on expatriate workforce ‐ Lack of training in local nurses
2	Abhicharttibutra et al., 2017. Thailand	To describe policy‐making processes to increase nursing capacity and improve nursing education quality	Key informants,	A qualitative study and document examination	‐ Policy formulation, policy implementation and policy modification
3	Adams et al., 2021 United Kingdom	To identify factors that affect the retention of mental health nursing workforce	23 studies were reviewed and assessed	Systematic search	‐ Individual characteristics, working conditions, training and skills and work environment
4	Aeschbacher & Addor, 2018 Switzerland	To identify nurses' working conditions and to explore potential to improve human resource management.	nurses who worked between 1970 and 2014	Retrospective longitudinal cohort study.	‐ Nurses experience flexibility in work hours ‐ Nurses experience more stress and aggression due to overwork.
5	Appiagyei et al., 2014. Kenya	To factor that influences training institutions' capacity to produce nurses	Nurse educators and/or administrators	Quantitative and qualitative interviews	‐ More student enrolment leads to congestion in clinical sites, inadequate classroom space, transport and accommodation.
6	Aluko et al., 2019 Nigeria	To describe the capacity of manpower and reasons for staff shortage	127 health in Nigeria	Mixed‐methods research design.	Shortage of manpower, disproportional skilled ratio, no framework for staff recruitment
7	Aiyub et al., 2013 Fiji	To report on vacant nursing position and movement among nurses.	Nurses in Fiji's schools of nursing from 2001–2010.	Descriptive study involving a retrospective record review	‐ Reasons for moving out from the nursing workforce was resignation of the nurses.
8	Alameddine et al., 2017 Germany	To identify trends in job satisfaction and job stickiness of nurses in 1990–2013	997 Female nurses.	Survey	‐ Decline in job satisfaction. ‐ Job stickiness of German nurses increased from 83 to 91%, while their job satisfaction declined by 7.5%.
9	Alreshidi et al., 2021 Saudi Arabia	To explore factors attributes to turnover among foreign nurses	639 foreign nurses	Quantitative based cross‐sectional descriptive study design	‐ Professional growth and development, leadership style, management, wage and benefit, workload.
10	Alshmemri et al., 2013 Saudi Arabia	To explore the job satisfaction levels of nurses working	272 Saudi national nurses	Mixed‐method design	‐ Female nurses not satisfied with their jobs as male nurses in Saudi Arabia ‐ Experience nurses of 4–10 years in nursing were also dissatisfied.
11	Amadi, 2015 Southern California	To investigate the effects of the nursing shortage and patients' health‐related outcomes	18 healthcare leaders	Qualitative method	Increasing nurses workload, absenteeism, nurse and patient dissatisfaction, lack of leadership support and challenges of budget
12	Atefi et al., 2016 Malaysia	To explore factors related to job satisfaction and job dissatisfaction	46 nurses	Qualitative study design	Nurses' personal values and beliefs; work environment factors and motivation factors.
13	Batran, 2019 Saudi Arabia	To identify the sources of stress among nurses	213 nurses in intensive care unit	Quantitative study	‐ Workload, lack of human resources and support ‐ Headache, back pain and mental health problem lead to nervousness and exhaustion.
14	Bradley et al., 2015 Malawi	To look more deeply at the impact on these cadres	84 health workers	Qualitative research design,	‐ Intention to leave current post.; too few staff, too many patients; lack of clinical officers causes physical and psychological consequences for staff to deliver quality care.
15	Buchan et al., 2015 Columbia	To examine metrics and policies regarding nurse workforce	Data collected from workforce policy	Quantitative study	‐ Nurses migration, frozen pay, ageing workforce drive projected shortages.
16	Chan et al., 2013 Hong Kong	To present findings on nurses intention to leave their employment	31 papers were synthesized	Electronic databases	‐ Job satisfaction, burnout and demographic factors, work environment, commitment, demand of work and social support.
17	Drennan and Ross (2019) South London	To investigate nurse workforces and shortages	14 Board Directors of nursing & Human Resources	Quantitative study.	“Pulled” nurses to other posts and factors that “pushed” nurses to leave.
18	Gibson & Greene, 2020 United States	To examine nursing home characteristics associated with shortages of staffing.	13,445 facilities	Facility‐level data, released 31 July 2020, from the Nursing Home COVID‐19 Public File,	30.2% of facilities 1 week of staffing shortage and 46.5% of facilities lacked staff.
19	Gray et al., 2018 Midland	To explore nurses and healthcare assistants' satisfaction with team working	127 Registered nurses and healthcare assistant	Quantitative study	‐ Dissatisfied with ability to carry out duties
20	Guo, 2017 Hunan Province, China	To investigate the prevalence of burnout on nurses	1,061 nurses in the hospital	A cross‐sectional survey design	‐ Nurses experienced severe burnout ‐ Shift work was the risk of the three metrics of burnout.
21	Heijden et al., 2019 Netherlands	To investigate whether burnout mediates the relationship with occupational turnover intention.	Registered nurses in the Netherlands	Quantitative study	‐ Perceived stress causes burnout levels that lead to a higher occupational turnover intention.
22	Hudgins, 2016 Victoria Australia	To explore the retirement decisions	295 aged care workers	Quantitative study	Excessive workload, pay and conditions associated with job satisfaction.
23	Hung & Lam, 2020 Hong Kong	To identify factors that contribute to occupational turnover from the clinical duties	18 Registered nurses	A qualitative descriptive study	‐ Job dissatisfaction due to stressed work environment, low motivation due to limited career opportunities and inadequate communication due to ineffective leadership.
24	Jarrar et al., 2018 Malaysia	To examine the effects of patient‐centeredness on nursing shortage	1,055 nurses	Quantitative study	Patient‐centeredness alleviates the negative associations of nursing shortage on the outcomes of care.
25	Kakemam et al., 2019 Iran	To determine nurses' occupational stress and associated risk	5,422 nurses	Cross‐sectional survey.	‐ Job was stressful. ‐ Shift work issues, staffing, pay, workplace discrimination, management, policy and excessive workloads.
26	Ke & Stocker, 2019 Taiwan	To explore new nurses' processes of growth in workplace	20 newly registered nurses	A qualitative study	‐ Most nurses (*n* = 18/20) went through these three stages and continued to work in nursing at follow‐up 2 years later.
27	Kurjenluoma et al., 2017 Finland	To describe the workplace stress, job satisfaction and practice environment.	577 nurses	The cross‐sectional data from September 2014 to January 2015	Nurses experienced stress and were satisfied with their job and their practice environment.
28	Leineweber et al., 2016 Sweden	To investigate nurse practice environment and work schedule that influenced the intention to leave the profession due to dissatisfaction.	23,076 registered nurses from 2020 units in 384 hospitals in 10 European countries	Cross‐sectional design.	Intention to leave the workplace was due to dissatisfaction.
29	LeVasseur & Qureshi, 2019 Hawaii	To provide an overview of nursing workforce in relation to population demographics, healthcare needs and gaps.	15,574 Registered nurses	Nursing Institution Sampling Method:	Shortage of nursing is 3,311 in the same year.
30	Liu et al., 2018 China	To investigate the preferences of nursing students when choosing a job.	554 final‐year undergraduate nursing students	A discrete choice experiment (DCE)	‐ 445 have a higher preference for a job with a higher monthly income
31	Marć et al., 2019 Poland	To address selected determinants of the nursing shortage in face of ageing	Nurses and Midwives	National listings and strategic documents	Shortage due to ineffective planning and use of available resources, poor recruitment and undersupply of new staff
32	Marufu et al., 2021 United Kingdom	To update factors affecting nurses intention to leave or stay in their profession	Primary study source from December 2018	5 online databases searched	‐ Nursing leadership and management, education and career advancement, work environment, professional issues and personal influence.
33	Masenyani et al., 2018 South Africa	To investigate the effects of absenteeism on nurses	361 nurses	A quantitative descriptive research	‐ Absenteeism has an effect on nurses' psychological and professional well‐being and the quality of patient care from psychological stress, low morale of nurses and increased workload.
34	Matsuo et al., 2021 Tokyo	To investigate the influence of work‐life balance sense of coherence or intention to leave	2,239 nurses	6‐month prospective cohort design	‐ Higher intentions to leave, work = life balance reduce nurses desire to quit their profession.
35	Mehdaova, 2017 Seattle, Washington	To explore the strategies healthcare leaders use to overcome a nursing shortage.	18 healthcare leaders	Qualitative design	‐ Increasing workload, absenteeism, nurses, patients' dissatisfaction and lack of leadership support and challenges of budget
36	Nantsupawat et al., 2017 Thailand	To investigate how work environment affects job dissatisfaction, burnout	1,351 nurses	Cross‐sectional survey	‐ Job dissatisfaction, burnout and nurses intention to leave their profession
37	Otto et al. (2019) Germany	To analyse whether nursing staff show work‐related burdens	242 nurses aged between 17 and 64 years	This quantitative study	All nurses require additional stress management. And need health promotion programmes to be implemented during the working time at the work setting
38	Park & Yu, 2019 Republic of Korea	To review effectiveness in reducing nursing shortages.	2,151 initial candidates	Systematic review to identify shortages of nurses via four electronic databases from January 23–31, 2019.	Various policies had been implemented worldwide to combat nursing shortages
39	Purohit & Vasava, 2017 India	To explain the concept of role stress and assesses the role stress	84 auxiliary nurse midwives (ANMs)	A Quantitative study (Survey)	‐ Role overload and role stagnation were the important role stressors for ANMs.
40	Seitovirta et al., 2017 Finland	To identify meaningful types of rewards and the consequences of rewards	20 registered nurses	A cross‐sectional, qualitative interview study.	Rewards: ‐ Financial compensation and benefits, Work‐Life balance, Work content, Professional development, Recognition and Supportive leadership.
41	Souza et al., 2017 Brazil	To describe and analyse the influence of economic and political models on the nursing hospital work process	34 nursing team workers	Qualitative descriptive research	‐ Human resources and material inadequacies that harm ‐ Wage decrease that causes the need for second jobs and work overload.
42	Sirisub et al., 2019 Thailand	To estimate RNs who intend to extend their working life and analysed the associations between general characteristics and quality of work life	3,629 RNs in the age group 55–59 years	A cross‐sectional study between October 2016 and April 2017	‐ Moderate or good working resources were the factors affecting intention to extend working life.
43	Uthaman et al., 2016 Australia	To determine evidence on older nurses, factors that promote or deter retirement	Reviews published between 2004 and 2015	Qualitative study	‐ Challenges are with ageing process, use of new technologies and shift work. ‐ Factors associated with retention are financial security
44	Varasteh et al., 2021 Iran	To explore the factors affecting nurses intention to leave or stay in their profession	16 nurses	Qualitative study	‐ Fear of family infected with Covid 19 ‐ Fear of PPE shortage ‐ Organizational factors
45	Wazqar, 2017 University, Saudi Arabia	To explore work stress and its sources among nurses	14 oncology nurses between October and December 2016.	Qualitative descriptive study	‐ Workload & staff shortage, emotional demand, lack of social support, language barriers and lack of respect from patients and family members.
46	Yang et al., 2017 China	To examine the work pressure and associated factors influencing the nurses' intent to leave	800 licensed nurses	A cross‐sectional questionnaire‐based survey	‐ Nurses' desire to leave the profession due to work pressure and workload
47	Yahyaei et al., 2022 United Kingdom	To synthesis and evaluate factors that effect intention to stay in the work environment	4,968 studies were screened	Systematic Review	‐ Personal and professional indicator, organization profile, work environment and patient‐related.
48	Yun et al., 2010 China	To assess whether there is a nursing shortage	1,543,257 Registered Nurses	Quantitative method	Changes in political structure, technology, globalization and nurse export to other countries lead to the shortage of nurses.

## 4 RESULTS

All the studies were published during 2010–2022. Table 2 shows that most studies were quantitative studies and most were conducted in Asia, European Countries, the United States of America and the rest from Africa, the Middle East, the United Kingdom and in the South Pacific. Of the total of 48 studies, 21 studies were conducted in Asia/Southern Asia and Western Asia (3 in Thailand, 5 in Saudi Arabia, 4 in China, 2 in Hong Kong, 2 in Malaysia, 1 in India and 2 in Iran, 1 Korea, 1 in Japan and 1 in Taiwan). Seven studies were conducted in the United States of America/Southern, Northern and eastern (1 in the United States, 1 in Southern California, 1 in Hawaii, 1 in Columbia, 1 in Midland, 1 in Washington and 1 in Brazil). Nine studies were conducted in Europe (2 in Germany, 2 in Finland and one each in Switzerland, London, Netherlands, Sweden and Poland). Four studies were conducted in Africa (one each in Kenya, Nigeria, Malawi and South Africa), three studies in the United Kingdom and three from the Pacific Islands (2 in Australia and 1 in Fiji).

**TABLE 2:** General characteristics of studies

Variable	Number	Percentage
Types of studies		
Quantitative	26	54.16
Mixed‐method	9	18.75
Qualitative	13	27.08
Region of studies conducted		
Asia	21	43.75
United States of America	7	14.58
European	9	18.75
Africa	4	8.33
United Kingdom	3	6.25
Middle East	1	2.08
South Pacific	3	6.25

From these 48 studies, the factors were identified to be related to the shortage of nursing workforce and were summarized and categorized into 4 themes: Policy and planning Barriers, Barriers to Training and enrolment, Factors causing staff turnover and Issues that affect nurses and patient health‐related outcomes.

### 4.1 Policy and planning barriers

Ten (20.83%) articles revealed that ineffective policies regulations and strategies, poor policy (Alameddine et al., 2017; Drennan & Ross, 2019; Seitovirta et al., 2017), poor planning and human resource planning, inadequate workforce planning and recruitment (Alreshidi et al., 2021; Aluko et al., 2019; Batran, 2019; Marć et al., 2019; Marufu et al., 2021; Purohit & Vasava, 2017), incompetent implementation plan and continue change of government officers (Park & Yu, 2019; Yun et al., 2010) and lack of leadership management (Souza et al., 2017) were main factors affecting shortage in nursing workforce. Furthermore, 7 (14.6%) articles supported that increased workload (Batran, 2019; Hudgins, 2016), unattractive working conditions, poor working conditions (Hung & Lam, 2020; Kakemam et al., 2019; Nantsupawat et al., 2017) and inadequate support affects nursing shortage and nurses' turnover (Alshmemri et al., 2013; Chan et al., 2013; Mehdaova, 2017). Five articles mentioned that barriers in policy and planning lead to nurses receiving poor salary, low level of job satisfaction, increasing ageing population and nurses to replace them and poor incentives for nurses (Alameddine et al., 2017; Hudgins, 2016; Sirisub et al., 2019; Souza et al., 2017; Uthaman et al., 2016).

### 4.2 Barriers to training and enrolment

Five (10.4%) articles revealed that decreasing nurse enrolment and lack of training for new nursing intakes are the main barriers that affect nursing shortage. Some training barriers are not enough spaces for training, special classroom, dormitories and clinical sites for practice (Aeschbacher & Addor, 2018; Alameddine et al., 2017; Amadi, 2015; Appiagyei et al., 2014; LeVasseur & Qureshi, 2019). Two (4.2%) articles revealed that importing nurses from other countries rather than train local nurses also has significant effects on nursing workforce (Aboshaiqah, 2016; Yun et al., 2010).

### 4.3 Factors causing nursing staff turnover

There are factors that cause nurses turnover, which then contributed to the shortage in nursing workforce. 21 (43.8%) articles revealed that professional vision towards nurses, lack of social support, work overload and low‐level job satisfaction are factors that cause staff turnover (Adams et al., 2021; Aeschbacher & Addor, 2018; Aiyub et al., 2013; Alameddine et al., 2017; Alshmemri et al., 2013; Chan et al., 2013; Drennan & Ross, 2019; Gray et al., 2018; Heijden et al., 2019; Hung & Lam, 2020; Ke & Stocker, 2019; Leineweber et al., 2016; Liu et al., 2018; Masenyani et al., 2018; Nantsupawat et al., 2017; Purohit & Vasava, 2017; Sirisub et al., 2019; Uthaman et al., 2016; Varasteh et al., 2021; Yahyaei et al., 2022; Yang et al., 2017). Another 10 (20.83%) articles confirmed that poor salaries or no changes to salary and poor working conditions are some of the contributing factors to nurses' turnover (Alameddine et al., 2017; Alreshidi et al., 2021; Chan et al., 2013; Hudgins, 2016; Kakemam et al., 2019; Marufu et al., 2021; Mehdaova, 2017; Park & Yu, 2019; Sirisub et al., 2019; Souza et al., 2017) and four (8.3%) articles confirmed that older nurses left work before their retirement age due to health conditions and limitation on new technology by older nurses are factors causing turnover (Buchan et al., 2015; Kurjenluoma et al., 2017; Sirisub et al., 2019; Uthaman et al., 2016).

### 4.4 Nurses' stress and burnout

There are major issues that affect nurses due to nursing workforce shortage, which contributes to nurse and patient health outcome. 14 (29.2%) articles revealed increased stress, burnout and psychosomatic disorders such as back and shoulder pain, anger and worry due to overwork (Aeschbacher & Addor, 2018; Batran, 2019; Bradley et al., 2015; Guo, 2017; Heijden et al., 2019; Hung & Lam, 2020; Kakemam et al., 2019; Matsuo et al., 2021; Otto et al., 2019; Purohit & Vasava, 2017; Seitovirta et al., 2017; Varasteh et al., 2021; Wazqar, 2017; Yang et al., 2017). The effects of shortage not only affect the nurses but also their family and social their social relationship as well. These have a significant impact on nurses, which affects the patient due to no early detection of patient complication, insufficient care provided to the patient and patient safety.

## 5 DISCUSSION

The shortage of the nursing workforce is a chronic issue that needs to be addressed effectively. Prompted by the findings on the nursing shortage, it impacted the health service delivery throughout the population (Ministry of Health (MOH), 2018). Although strategies have been implemented in the past to address the issues, the shortage of nursing is still evident. The aim of this study is to explore the healthcare professional's perception on factors affecting the shortage of nursing workforce and its impact on registered nurses in hospitals. The current study findings have reported the factors that influence the shortage in the nursing workforce and its impact on the Registered nurses.

In this systematic review, results show that ineffective policies regulations and strategies, poor policy, poor planning and human resource planning, inadequate workforce planning and recruitment, incompetent implementation plan and continue change of government officers and lack of leadership management were some main factors affecting shortage in nursing workforce (Abhicharttibutra et al., 2017; Alreshidi et al., 2021; Amadi, 2015; Buchan et al., 2015; LeVasseur & Qureshi, 2019; Marć et al., 2019; Marufu et al., 2021; Park & Yu, 2019; Yun et al., 2010). Increased workload, unattractive working conditions, poor working conditions and inadequate support affects nursing shortage as nurses tend to leave their job (Batran, 2019; Hung & Lam, 2020; Kakemam et al., 2019; Nantsupawat et al., 2017; Varasteh et al., 2021). Results also show that barriers in policy and planning lead to nurses receiving poor salary, low level of job satisfaction, increasing ageing population and nurses to replace them and poor incentives for nurses (Alameddine et al., 2017; Alreshidi et al., 2021; Amadi, 2015; Buchan et al., 2015; Hudgins, 2016; Jarrar et al., 2018; Kakemam et al., 2019; Marć et al., 2019; Marufu et al., 2021; Sirisub et al., 2019; Souza et al., 2017; Uthaman et al., 2016).

The results for barriers to training and enrolment also found that decreasing nurse enrolment and lack of training for new nursing intakes are the main barriers that affect nursing shortage. Some training barriers are not enough spaces for training, special classroom, dormitories and clinical sites for practice, which results in the decreased number of intakes at a time (Aeschbacher & Addor, 2018; Alameddine et al., 2017; Appiagyei et al., 2014; LeVasseur & Qureshi, 2019). Furthermore, the continuous import of expatriate nurses from other countries rather than training local nurses also has significant effects on nursing workforce (Aboshaiqah, 2016).

In this review, results show that professional vision towards nurses, lack of social support, work overload and low‐level job satisfaction are factors that cause nursing staff turnover (Aeschbacher & Addor, 2018; Alameddine et al., 2017; Alshmemri et al., 2013; Atefi et al., 2016; Chan et al., 2013; Drennan & Ross, 2019; Gray et al., 2018; Heijden et al., 2019; Hung & Lam, 2020; Ke & Stocker, 2019; Leineweber et al., 2016; Liu et al., 2018; Masenyani et al., 2018; Nantsupawat et al., 2017; Purohit & Vasava, 2017; Sirisub et al., 2019; Uthaman et al., 2016; Varasteh et al., 2021; Yang et al., 2017). Furthermore, results show that poor salaries or no changes to salary and poor working conditions as well as older nurses leave work before their retirement age due to health conditions and limitations on new technology by older nurses, which results in nurses turnover (Adams et al., 2021; Aeschbacher & Addor, 2018; Alameddine et al., 2017; Hudgins, 2016; Kakemam et al., 2019; Mehdaova, 2017; Park & Yu, 2019; Sirisub et al., 2019; Souza et al., 2017; Uthaman et al., 2016; Yahyaei et al., 2022).

Furthermore, increased stress, burnout and psychosomatic disorders such as back and shoulder pain, anger and worry due to overwork not only affects nurses but also has an impact on their family and social relationship when nurses have to work for more hours (Aeschbacher & Addor, 2018; Batran, 2019; Guo, 2017; Heijden et al., 2019; Hung & Lam, 2020; Kakemam et al., 2019; Matsuo et al., 2021; Otto et al., 2019; Purohit & Vasava, 2017; Seitovirta et al., 2017; Varasteh et al., 2021; Wazqar, 2017; Yang et al., 2017). These result in patient health outcomes due to no early detection of patient's complication, insufficient care and patient's safety (Batran, 2019; Kakemam et al., 2019; Masenyani et al., 2018; Seitovirta et al., 2017; Wazqar, 2017; Yang et al., 2017).

## 6 CONCLUSION

The worldwide shortage of nursing workforce is one of the obstacles in providing quality healthcare services to the population to improve health and well‐being and to achieve universal coverage. The inadequate supply of nurses has created many negative impacts on patient health‐related outcomes, as well as challenges to fight diseases and improves health. This also causes an increased workload on nurses and later results in decreasing the quality of nursing care, threaten the safety of patients and increase the workforce mental health issues. The study synthesized the findings of forty‐two studies. The reasons for the shortage in nursing workforce and the effects were influenced by many factors that are summarized and categorized into barriers to policy and planning, training and enrolment barriers, nursing staff turnover and the impacts on the nurses and patient health outcomes.

### 6.1 Implications for nursing management

There are factors that significantly contributed to the nursing shortage worldwide such as barriers in policy and planning, training and enrolment and staff turnover due to workload and job dissatisfaction. Intervention to address these factors promptly and appropriately is crucial or quality nursing care will continue to be affected and the primary goal to improve and protect the health of the individual will continue to be a challenge. The findings of this study can be helpful to key health professionals at the decision‐making level or policymakers to resolve the nursing workforce shortage in the future. Further research is needed to determine the in‐depth effects of nursing workforce shortage on patients and interventions to address it more effectively.

## AUTHOR CONTRIBUTIONS

AT and MM conceived the initial idea of the paper. AT wrote the first draft. MM critically revised the initial drafts. All authors were involved in the revision of the final draft of the paper and approved the final manuscript.

## CONFLICT OF INTEREST

The authors declare no conflict of interest.

## ETHICAL APPROVAL

This systematic review study does not need ethical approval as it retrieved and analysed the previous published studies in which informed consent was obtained by primary investigators.

## Data Availability

The data sets used and/or analysed during the current study are available from the corresponding author upon request.
